# 

**Published:** 1892-09

**Authors:** 


### PAMPHLETS RECEIVED.

The Caustic Treatment of Cancer. By Daniel Lewis, M. D., New York City.

The Teachings of Experience as to the Treatment of Pneumonia. By Boardman Reed, M. D., Atlantic City, N. J.

Drug Habituation. By L. W. Baker, M. D., Baldwinville, Mass.

Myelitis in Posterior Spinal Sclerosis. By J. T. Eskridge, M. D., Denver, Colorado.

Ataxia. By J. T. Eskridge, M. D., Denver, Colorado.

New Method of Skin Grafting. By P. A. Morrow, M. D., of New York.

Differential Points in the Diagnosis of Syphilis and Tuberculosis, with Cases. By P. A. Morrow, M. D., New York.

Successful Case of Trephining for Cerebral Hemorrhage. By Emory Lanphear, M. D., Kansas City, Mo.

Treatment of Penetrating Wounds of the Abdomen. By Emory Lanphear, M. D.

Bloodless Amputation at the Hip. By Emory Lanphear, M. D.

Experiments in Pneumonotomy and Pneumonectomy. By DeForest Willard, M. D., Philadelphia.

Intra-Thoracic Surgery; Bronchotomy through Chest-wall for Foreign Bodies Impacted in the Bronchi. By DeForest Willard, M. D.

Surgical and Mechanical Treatment of Deformities Following Infantile Spinal Paralysis. By DeForest Willard, M. D.

Pathological Anatomy of Acute Arsenical Poisoning. By Ludwig Hektoen, M. D., Chicago.

Rupture of the Aortic Valves, with Demonstration of Specimen. Aneurisms of Right Auricular Appendix. By Ludwig Hektoen, M. D., Chicago.

Pelvic Inflammation in Women: A Pathological Study. By Dr. W. W. Potter, Buffalo, N. Y.

Asepsis and Antisepsis in the Lying-in Chamber. By Dr. W. W. Potter, Buffalo, N. Y.

Report on Abdominal and Pelvic Surgery, Including Thirty-two Successful Cases of Laparotomy. By William H. Wathen, M. D., Louisville.

Gynecological Technique. By Howard A. Kelley, M. D., John Hopkins University, Baltimore.

